# Perceived relative harm of heated tobacco products and electronic cigarettes and its association with use in smoke-free places: A cross-sectional analysis of Korean adults

**DOI:** 10.18332/tid/145699

**Published:** 2022-02-21

**Authors:** Choon-Young Kim, Kiheon Lee, Cheol Min Lee, Sungroul Kim, Hong-Jun Cho

**Affiliations:** 1 Department of Family Medicine, Healthcare System Gangnam Center, Seoul National University Hospital, Seoul, Republic of Korea; 2 Department of Family Medicine, Seoul National University Bundang Hospital, Seongnam, Republic of Korea; 3 Department of Family Medicine, Seoul National University College of Medicine, Seoul, Republic of Korea; 4 Department of Environmental Health Sciences, Soonchunhyang University, Asan, Republic of Korea; 5 Department of Family Medicine, Asan Medical Center, University of Ulsan College of Medicine, Seoul, Republic of Korea

**Keywords:** secondhand smoke, electronic nicotine delivery devices, non-cigarette tobacco products

## Abstract

**INTRODUCTION:**

Electronic cigarettes (e-cigarettes) and heated tobacco products (HTPs) are often considered to be less harmful and safer than combustible cigarettes (CCs). As a result, numerous tobacco product users opt to use e-cigarettes or HTPs as a safer alternative, though the safety of these products is not fully warranted. The present study aimed to assess the various attitudes towards e-cigarettes and/or HTPs among Korean tobacco product users and their associations with the practical use of e-cigarettes and/or HTPs in private or smoke-free public places.

**METHODS:**

A cross-sectional study using self-administered questionnaires was conducted from March 2019 to July 2019 on 2971 adult tobacco product users. Attitude towards e-cigarettes and/or HTPs, as well as the relative harm perceptions, in association with their practical use in private or smoke-free areas, were also analyzed.

**RESULTS:**

Among those surveyed, 46.8% were exclusive users (CC-only smokers 23.5%, e-cigarette-only users 10.7%, HTP-only users 12.7%), and 47.6% were poly-users. Compared with non-e-cigarette or non-HTP users, current e-cigarette or HTP users perceived e-cigarettes or HTPs as less harmful than CCs and they were more acceptable to e-cigarettes or HTPs being used indoors. Their positive attitudes were associated with their more frequent use at home or in their car. Less number of participants supported that the government should regulate e-cigarettes or HTPs in the same way as CCs, their attitude being associated with more frequent use in smoke-free public places.

**CONCLUSIONS:**

E-cigarettes or HTPs users have more positive attitudes toward their tobacco products than non-e-cigarette or non-HTP users. Those with more positive attitudes toward e-cigarettes or HTPs are closely related to their use in smoke-free places.

## INTRODUCTION

Tobacco use is the leading cause of preventable death in the world, killing more than 8 million people each year^1,2^. Although smoking cessation is one of the most effective ways to save lives, only 4% of attempts to quit tobacco succeed without professional support. Due to the difficulty in quitting smoking, and the promotion that electronic cigarettes (e-cigarettes) and heated tobacco products (HTPs) pose less health risks than combustible cigarettes (CCs) has resulted in these products being readily embraced by the smoking community, especially by those who failed to quit smoking^3^.

As a result, IQOS, the leading brand of HTPs (by Philip Morris International), accounted for 2.2% of the total sales of tobacco products in its first year of launch (2017), and the figure rose to 10.5% within two years of launch in South Korea^4,5^. Recently, the U.S. Food and Drug Administration (FDA) issued more stringent regulations on e-cigarettes due to increased adolescent use^6^, and the FDA authorized the marketing of IQOS as modified risk tobacco products^7^. Recent studies on the effects of IQOS on risk perceptions and behavioral interventions showed that there were often significant omissions in consumers’ perceptions of modified risk claims^8^. As such, the FDA’s ‘reduced-exposure’ orders may confuse tobacco users, and consumers may fail to understand the difference between reduced-exposure and reduced-harm claims^9^. There is little evidence regarding the long-term health effects of e-cigarettes and HTPs^10^. However, the extensive and proliferating advertising of many tobacco companies has led the public, especially current tobacco users, to have a positive impression of e-cigarettes and HTPs. Although many smokers understand that HTPs are not risk-free, the prevalent belief that HTPs pose fewer health risks than CCs encourages them to use HTPs^11^. A recent study of Korean adults found that HTP-only users and poly-users of tobacco products were more likely to perceive HTPs as less harmful than CCs than CC-only smokers^11^. Furthermore, a recent Japanese study showed that a considerable number of people used HTPs in places where tobacco smoking was not allowed^12^.

The present study aimed to assess the various attitudes towards e-cigarettes and/or HTPs among Korean tobacco product users and their associations with the practical use of e-cigarettes and/or HTPs in private or smoke-free public areas.

## METHODS

### Sample

Considering the small sample size of some groups in the general population (e.g. dual users of e-cigarettes and HTPs), we targeted a convenience sample aged ≥19 years from March 2019 to July 2019^13^. For effective analysis, the minimum sampling number was set at 300 adults per group, excluding non-tobacco users. The survey was conducted using a structured questionnaire in two ways. Firstly, the online survey was performed using a sample from a panel managed by a central Korean research agency, Gallup Korea (https://www.gallup.co.kr/), comprising 1.1 million members as of February 2019. Secondly, another face-to-face interview was conducted using a tablet-assisted personal interview at five health screening centers and one university. Participants were initially screened for classification into groups by asking a series of questions about each of the following three types of tobacco products: CCs, e-cigarettes, and HTPs. As part of the preamble to questions on tobacco use, these questions were characterized with detailed descriptors and pictures of e-cigarettes and HTPs to prevent confusion with other tobacco products. Because HTP adopted the official name of e-cigarettes (cigarette-like e-cigarette), we added the specific brand name of HTPs on sale in South Korea to prevent confusion with e-cigarettes. After categorizing the participants into one of eight groups (non-tobacco users, CC-only smokers, e-cigarette-only users, HTP-only users, dual users of CC and e-cigarette, dual users of e-cigarette and HTP, dual users of CC and HTP, and triple users), they were given a detailed questionnaire. All participants received financial incentives equivalent to 3000 Korean Won (KRW) for the online panel or 10000 KRW (about US$8.4) for offline participants.

### Measures

Demographic characteristics including sex, age, educational level, household income, and marital status were collected. Ages were categorized as follows: <30, 30–39, 40–49, or ≥50 years. Education was categorized as high school level or lower, college level, or postgraduate level. Household income in KRW was classified as follows: <3 million, 3 million–4999999, or ≥5 million. Marital status was categorized as married or living with a partner, separated, widowed, divorced, never married, and with a spouse or without a spouse. Alcohol consumption frequency was categorized as ≤1 per month, 2–4 per month, or at least weekly. Subjective health status was categorized as good, fair, or bad, and chronic cough for more than 3 months was asked (Yes/No).

*Types of tobacco use*

Cigarette smoking was assessed by asking the following question: ‘Do you currently smoke cigarettes every day, some days, or not at all?’. Current cigarette smokers were those who responded as every day or some days, with lifetime use of 100 or more cigarettes. E-cigarette use was assessed by asking the following questions: ‘Have you ever used e-cigarettes in your life?’ (Yes/No) and ‘Did you use e-cigarettes in the past 30 days?’ (Yes/No). Current e-cigarette users were those who had used e-cigarettes in their lifetime and the past 30 days. HTP use was assessed through asking the following questions: ‘Have you ever used HTPs (e.g. IQOS, Glo, and Lil) in your life?’ and ‘Do you currently use HTPs every day, some days, or not at all?’. Current HTP users were those who responded as every day or some days^14^. Single-use was defined as use of only one type of tobacco product (e.g. exclusively CCs, e-cigarettes, or HTPs), whereas multiple product use was determined by using two (dual use; e.g. both e-cigarette and HTP) or three (triple use) types of tobacco products.

*Attitude towards e-cigarettes and HTPs*

For all participants, the perceived harmfulness of e-cigarettes or HTPs was assessed by asking: ‘Do you think e-cigarettes or HTPs are more harmful than CCs, less harmful, or are they equally harmful to health?’ (options: ‘less’, ‘equally’, ‘more’, and ‘don't know’). Harmfulness of exposure to secondhand aerosols from e-cigarettes or HTPs compared to CCs was assessed in a similar way. Acceptability toward indoor use of e-cigarettes or HTPs was assessed by: ‘In your opinion, how acceptable or unacceptable is it to use e-cigarettes or HTPs in indoor spaces?’ (options: ‘acceptable’, ‘neutral’, ‘unacceptable’ and ‘don't know’). Attitude towards regulations against e-cigarettes or HTPs was assessed by asking: ‘In your opinion, do you agree that e-cigarette or HTP should be regulated as CCs?’ (options: ‘unsupportive’, ‘neutral’, ‘supportive’, and ‘don't know’). Current users of e-cigarettes or HTPs were asked whether they used the respective products in the last 30 days ‘in public places where CC smoking is not allowed?’ (Yes/No), ‘inside your home?’ (Yes/No), and ‘in your car?’ (Yes/No).

### Statistical analysis

To analyze attitudes towards e-cigarettes, the participants were reclassified into three groups according to the patterns of using e-cigarettes: non-tobacco users, non-e-cigarette users (e.g. CC-only smokers, HTP-only users, and dual users of CCs and HTPs), and e-cigarette-users (e.g. e-cigarette-only users, dual users of CCs and e-cigarettes, dual users of e-cigarettes and HTPs, and triple users). To analyze attitudes towards HTPs, the participants were reclassified into three groups according to the patterns of using HTPs: non-tobacco users, non-HTP users (e.g. CC-only smokers, e-cigarette-only users, and dual users of CCs and e-cigarettes), and HTP users (e.g. HTP-only users, dual users of e-cigarettes and HTPs, dual users of CCs and HTPs, and triple users).

Descriptive statistics using frequencies and percentages are presented separately, and the three groups are compared in pairs (non-tobacco users vs non-e-cigarette (or non-HTP) users; non-tobacco users vs e-cigarette (or HTP) users; non-e-cigarette (or non-HTP) users versus e-cigarette (or HTP users) using the chi-squared tests with Bonferroni correction for multiple comparisons (p<0.017).

To examine the association between the use of e-cigarettes or HTPs and the attitudes towards each product (harm perception, acceptability of use in indoor places, support for government regulation), we conducted multivariable logistic regression analysis after adjusting for sex, age, residential area, educational level, household income, marital status, alcohol consumption frequency, chronic cough (for more than three months), self-rated health status, survey mode, and one’s position regarding government regulations. When assessing the participants’ stance towards government regulations, however, the harm perception of each product was adjusted instead of their position. To determine the association between attitude and patterns of use among current users, we conducted a similar multivariable logistic regression analysis after adjusting for confounders as mentioned above. STATA 14.0 (College Station, Texas, USA) was used for statistical analysis, and p<0.05 was considered statistically significant.

## RESULTS

### General characteristics of study participants

The general characteristics of the 2971 participants included in the final analysis are summarized in Table 1. The mean age was 40.3 years. The proportion of participants aged <30 years was 20.9%, 30–39 years 27.4%, 40–49 years 28.4%, and those aged >50 years was 23.3%. Most of the participants were: male (75.6%), residents of the metropolis (63.2), and education level above college (82.6%). Sufferers of chronic cough (for ≥3 months) comprised less than 10% of the participants, and those who rated their health status as ‘bad’ were 12.4% of the participants.

**Table 1:** General characteristics of study participants (N=2971)

*Characteristics*	*Categories*	*n*	*%*
**Sex**	Male	2246	75.6
	Female	725	24.4
**Age** (years)	<30	622	20.9
	30–39	814	27.4
	40–49	843	28.4
	≥50	692	23.3
**Survey mode**	Offline	980	33.0
	Online	1991	67.0
**Residential area**	Metropolitan	1877	63.2
	Other	1094	36.8
**Education level**	≤ High school	518	17.4
	≥ College	2453	82.6
**Household income** (in 10000 KRW)	<500	1373	46.7
	≥500	1570	53.3
**Marital status**	With spouse	1705	57.4
	Without spouse	1266	42.6
**Type of tobacco use**	Non-tobacco use	167	5.6
	CC-only use	698	23.5
	e-cigarette-only use	316	10.7
	HTP-only use	377	12.7
	Dual use of CCs and e-cigarettes	374	12.6
	Dual use of e-cigarettes and HTPs	303	10.2
	Dual use of CCs and HTPs	393	13.2
	Triple use	343	11.5
**Alcohol consumption frequency**	≤1 per month	882	29.7
	2–4 per month	1051	35.4
	At least weekly	1038	34.9
**Chronic cough ≥3 months**	Yes	286	9.6
**Subjective health status**	Good	926	31.2
	Fair	1677	56.4
	Bad	368	12.4

KRW: 10000 Korean Won about US$8.4. CC: combustible cigarette. e-cigarette: electronic cigarette. HTP: heated tobacco product.

### Attitude toward e-cigarettes or HTPs

The participants’ attitudes towards e-cigarettes or HTPs were analyzed and are summarized in Tables 2 and 3. Multivariable analysis after adjustment for possible confounders showed that the number of participants who answered that e-cigarettes were less harmful than CCs was significantly higher in e-cigarette users than non-tobacco users (adjusted odds ratio, AOR= 4.4; 95% CI: 2.6–7.3, p<0.001). More respondents answered that e-cigarettes were more harmful than CCs among CC and HTP users (43.1%) and among non-tobacco users (62.9%). Similarly, regarding the risk of secondhand exposure, e-cigarette users answered that exposure to secondhand aerosol from e-cigarettes was less harmful than secondhand smoke from CCs (AOR=5.1; 95% CI: 3.1–8.6, p<0.001), unlike non-e-cigarette users and non-tobacco users. Regarding the indoor use of e-cigarettes, most respondents answered that e-cigarettes use in indoor places was unacceptable in all groups. Still, the rate was the lowest in e-cigarette users (55.8%) and highest in non-tobacco users (86.8%) (AOR=11.8; 95% CI: 3.6–38.0, p<0.001). Concerning the policy that the government should regulate e-cigarettes in the same way as CCs, more respondents answered that they support the policy. At the same time, the rate was lowest in e-cigarette users (34.8%) and highest in non-tobacco users (71.3%) (AOR=2.5; 95% CI: 1.5–4.1, p<0.001). Harm perception and attitudes towards HTPs were like those towards e-cigarettes (Tables 2 and 3). Detailed data on the participant’s attitude toward e-cigarettes or HTPs in each group according to the response to tobacco product use were analyzed and are summarized in the Supplemental file Tables S1 and S2.

**Table 2:** Harm perception and attitudes towards electronic cigarettes or heated tobacco products according to the tobacco product users (N=2971)

	*Attitudes towards e-cigarettes*				*Attitudes towards HTPs*			
	*Total users (n=2971)*	*Non-tobacco users (n=167)*	*Non-e-cigarette users (n=1468)*	*E-cigarette users (n=1336)*	*Total users (n=2971)*	*Non-tobacco users (n=167)*	*Non-HTP users (n=1388)*	*HTP users (n=1416)*
**Harmfulness of e-cigarettes (or HTPs) relative to CCs**								
Less	785 (26.4)	22 (13.2)	265 (18.1)	498 (37.3)	770 (25.9)	16 (9.6)	248 (17.9)	506 (35.7)
Equally	787 (26.5)	20 (12.0)	359 (24.5)	408 (30.5)	768 (25.9)	21 (12.6)	342 (24.6)	405 (28.6)
More	1132 (38.1)	105 (62.9)	632 (43.1)	395 (29.6)	1211 (40.8)	108 (64.7)	637 (45.9)	466 (32.9)
Don’t know	267 (9.0)	20 (12.0)	212 (14.4)	35 (2.6)	222 (7.5)	22 (13.2)	161 (11.6)	39 (2.8)
**Harmfulness of exposure to secondhand aerosol from e-cigarettes (or HTPs) relative to secondhand smoking from CCs**								
Less	936 (31.5)	21 (12.6)	375 (25.5)	540 (40.4)	834 (28.1)	11 (6.6)	288 (20.8)	535 (37.8)
Equally	761 (25.6)	29 (17.4)	350 (23.8)	382 (28.6)	783 (26.4)	23 (13.8)	360 (25.9)	400 (28.2)
More	1030 (34.7)	96 (57.5)	547 (37.3)	387 (29.0)	1135 (38.2)	113 (67.7)	584 (42.1)	438 (30.9)
Don’t know	244 (8.2)	21 (12.6)	196 (13.4)	27 (2.0)	219 (7.4)	20 (12.0)	156 (11.2)	43 (3.0)
**Acceptability of e-cigarettes (or HTPs) use in indoor places**								
Acceptable	432 (14.5)	3 (1.8)	182 (12.4)	247 (18.5)	337 (11.3)	3 (1.8)	107 (7.7)	227 (16.0)
Neutral	573 (19.3)	12 (7.2)	246 (16.8)	315 (23.6)	588 (19.8)	8 (4.8)	223 (16.1)	357 (25.2)
Unacceptable	1800 (60.6)	145 (86.8)	909 (61.9)	746 (55.8)	1895 (63.8)	146 (87.4)	945 (68.1)	804 (56.8)
Don’t know	166 (5.6)	7 (4.2)	131 (8.9)	28 (2.1)	151 (5.1)	10 (6.0)	113 (8.1)	28 (2.0)
**Support for government regulation of e-cigarettes (or HTPs) in the same way as CCs**								
Unsupportive	706 (23.8)	24 (14.4)	283 (19.3)	399 (29.9)	650 (21.9)	22 (13.2)	228 (16.4)	400 (28.3)
Neutral	776 (26.1)	18 (10.8)	320 (21.8)	438 (32.8)	783 (26.4)	19 (11.4)	330 (23.8)	434 (30.7)
Supportive	1318 (44.4)	119 (71.3)	734 (50.0)	465 (34.8)	1394 (46.9)	118 (70.7)	724 (52.2)	552 (39.0)
Don’t know	171 (5.8)	6 (3.6)	131 (8.9)	34 (2.5)	144 (4.9)	8 (4.8)	106 (7.6)	30 (2.1)

A total of 2971 Koreans were sampled from March 2019 to July 2019. Results are expressed as number (%) and 3 groups were compared in pairs: Non-tobacco users vs non-e-cigarette (or non-HTP) users; Non-tobacco users vs e-cigarette (or HTP) users; Non-e-cigarette (or non-HTP) users vs e-cigarette (or HTP) users; using chi-squared tests with Bonferroni correction for multiple comparison (p<0.017). CC: combustible cigarette. e-cigarette: electronic cigarette. HTP: heated tobacco product.

**Table 3:** The association of tobacco product users with their attitudes towards electronic cigarettes or heated tobacco products (N=2971)

	*Attitudes towards e-cigarettes*			*Attitudes towards HTPs*		
	*Non-tobacco users (n=167)*	*Non-e-cigarette users (n=1468)*	*E-cigarette users (n=1336)*	*Non-tobacco users (n=167)*	*Non-HTP users (n=1388)*	*HTP users (n=1416)*
**Harmfulness of e-cigarettes (or HTPs) relative to CCs**						
Less harmful versus others	1 (Ref.)	1.4 (0.9–2.4)	4.4 (2.6–7.3)**	1 (Ref.)	1.8 (1.0–3.3)*	6.3 (3.5–11.2)**
**Harmfulness of exposure to secondhand aerosol from e-cigarettes (or HTPs) relative to secondhand smoking from CCs**						
Less harmful versus others	1 (Ref.)	2.1 (1.3–3.6)*	5.1 (3.1–8.6)**	1 (Ref.)	3.0 (1.5–6.0)**	9.9 (5.0–19.5)**
**Acceptability of e-cigarettes (or HTPs) use in indoor places**						
Acceptable versus others	1 (Ref.)	8.2 (2.5–26.9)**	11.8 (3.6–38.0)**	1 (Ref.)	4.7 (1.4–16.1)*	10.5 (3.3–33.7)**
**Support for government regulation of e-cigarettes (or HTPs) in the same way as CCs**						
Unsupportive versus others	1 (Ref.)	1.3 (0.8–2.2)	2.5 (1.5–4.1)**	1 (Ref.)	1.5 (0.9–2.5)	2.1 (1.3–3.5)*

A total of 2971 Koreans were sampled from March 2019 to July 2019. Results are expressed as AOR (95% CI) and p-values are calculated in pairs (less harmful, acceptable, or unsupportive, vs others), using multivariable analyses after adjusting for sex, age, residential area, educational level, household income, marital status, alcohol consumption frequency, chronic cough, subjective health, survey mode, and the support for government regulation. When assessing the support for government regulation, the harm perception of each product was adjusted instead of the support for government regulation. CC: combustible cigarette. e-cigarette: electronic cigarette. HTP: heated tobacco product. AOR: adjusted odds ratio. CI: confidence interval.

* p<0.05

** p<0.001.

### Association between attitude toward e-cigarettes or HTPs and the actual use in smoke-free places

The association between participants’ attitudes towards e-cigarettes or HTPs and the actual use in private and public places were analyzed and are summarized in Tables 4 and 5. Multivariable analysis after adjusting for possible confounders showed that the frequency of actual use of e-cigarettes at home, in cars, and in public places, was significantly higher among participants who answered that e-cigarettes were less harmful than CCs (home: AOR=2.0; 95% CI: 1.6-2.5, p<0.001; cars: AOR=1.4; 95% CI: 1.0–1.8, p=0.030; and public places: AOR=1.8; 95% CI: 1.4–2.4, p<0.001). Similarly, regarding the risk of secondhand exposure, the actual use of e-cigarettes at home, in cars, and in public places was significantly more frequent among participants who answered that exposure to secondhand aerosol from e-cigarettes was less harmful than exposure to secondhand smoking from CCs (home: AOR=2.4; 95% CI: 1.9–3.0, p<0.001; cars: AOR=1.6; 95% CI: 1.2–2.1, p=0.002; and in public places: AOR=1.4; 95% CI: 1.1–1.9, p=0.018). Regarding the indoor use of e-cigarettes, the actual use of e-cigarettes at home, in cars, and in public places was significantly more frequent among participants who answered that it is acceptable to use e-cigarettes in indoor places (home: AOR=3.6; 95% CI: 2.7–4.9, p<0.001; cars: AOR=2.4; 95% CI: 1.8–3.3, p<0.001; and public places: AOR=2.5; 95% CI: 1.8–3.5, p<0.001). Attitudes towards HTPs and actual use in smoke-free places were similar to those towards e-cigarettes (Tables 4 and 5). As for the policy that the government should regulate e-cigarettes in the same way as CCs, the actual use of e-cigarettes at home, in cars, and in public places was significantly more frequent among participants who answered that they were unsupportive for government to regulate e-cigarettes to the same level as CCs (home: AOR=1.5; 95% CI: 1.1–1.9, p=0.002; cars: AOR=1.6; 95% CI: 1.2–2.2, p=0.001; and public places: AOR=1.6; 95% CI: 1.2–2.2, p=0.002). Meanwhile, among participants who answered that they were unsupportive for the government to regulate HTPs at the same level as CCs, the actual use of HTPs in cars was significantly more frequent (home: AOR=1.3; 95% CI: 1.0–1.7, p=0.037; cars: AOR=1.5; 95% CI: 1.1–2.0, p=0.007). There was, however, no significant difference in the actual use of HTPs in public places among all participants.

**Table 4:** The actual use of electronic cigarettes or heated tobacco products in private or public places according to the harm perception and attitudes towards electronic cigarettes or heated tobacco products

	*Using e-cigarettes in private or public places (n=1336)*			*Using HTPs in private or public places (n=1416)*		
	*Home (n=593; 44.4%)*	*Car (n=276; 20.7%)*	*Public places (n=248; 18.6%)*	*Home (n=592; 41.8%)*	*Car (n=289; 20.4%)*	*Public places (n=246; 17.4%)*
**Harmfulness of e-cigarettes (or HTPs) relative to CCs**						
Less	273 (54.8)	120 (24.1)	117 (23.5)	262 (51.8)	127 (25.1)	116 (22.9)
Equally	165 (40.4)	85 (20.8)	60 (14.7)	157 (38.8)	81 (20.0)	61 (15.1)
More	139 (35.2)	64 (16.2)	65 (16.5)	159 (34.1)	77 (16.5)	65 (14.0)
Don’t know	16 (45.7)	7 (20.0)	6 (17.1)	14 (35.9)	4 (10.3)	4 (10.3)
**Harmfulness of exposure to secondhand aerosol from e-cigarettes (or HTPs) relative to secondhand smoking from CCs**						
Less	310 (57.4)	135 (25.0)	113 (20.9)	304 (56.8)	126 (23.6)	111 (20.8)
Equally	146 (38.2)	82 (21.5)	67 (17.5)	140 (35.0)	81 (20.3)	68 (17.0)
More	124 (32.0)	53 (13.7)	63 (16.3)	133 (30.4)	77 (17.6)	60 (13.7)
Don’t know	13 (48.2)	6 (22.2)	5 (18.5)	592 (34.9)	5 (11.6)	7 (16.3)
**Acceptability of e-cigarettes (or HTPs) use in indoor places**						
Acceptable	173 (70.0)	85 (34.4)	78 (31.6)	154 (67.8)	81 (35.7)	69 (30.4)
Neutral	166 (52.7)	76 (24.1)	65 (20.6)	172 (48.2)	79 (22.1)	74 (20.7)
Unacceptable	243 (32.6)	107 (14.3)	101 (13.5)	258 (32.1)	123 (15.3)	97 (12.1)
Don’t know	11 (39.3)	8 (28.6)	4 (14.3)	8 (28.6)	6 (21.4)	6 (21.4)
**Support for government regulation of e-cigarettes (or HTPs) in the same way as CCs**						
Unsupportive	198 (49.6)	104 (26.1)	95 (23.8)	182 (45.5)	100 (25.0)	82 (20.5)
Neutral	193 (44.1)	87 (19.9)	76 (17.4)	190 (43.8)	84 (19.4)	79 (18.2)
Supportive	185 (39.8)	79 (16.0)	71 (15.3)	205 (37.1)	100 (18.1)	78 (14.1)
Don’t know	17 (50.0)	6 (17.7)	6 (17.7)	15 (50.0)	5 (16.7)	7 (23.3)

A total of 2971 Koreans were sampled from March 2019 to July 2019. Results are expressed as number (%). CC: combustible cigarette. e-cigarette: electronic cigarette. HTP: heated tobacco product.

**Table 5:** The association between harm perception and attitudes towards electronic cigarettes or heated tobacco products and the actual use of electronic cigarettes or heated tobacco products in private or public places

	*Using e-cigarettes in private or public places (n=1336)*			*Using HTPs in private or public places (n=1416)*		
	*Home (n=593; 44.4%)*	*Car (n=276; 20.7%)*	*Public places (n=248; 18.6%)*	*Home (n=592; 41.8%)*	*Car (n=289; 20.4%)*	*Public places (n=246; 17.4%)*
**Harmfulness of e-cigarettes (or HTPs) relative to CCs**						
Less harmful versus others	2.0 (1.6–2.5)**	1.4 (1.0–1.8)*	1.8 (1.4–2.4)**	1.7 (1.3–2.1)**	1.4 (1.1–1.9)*	1.7 (1.3–2.3)*
**Harmfulness of exposure to secondhand aerosol from e-cigarettes (or HTPs) relative to secondhand smoking from CCs**						
Less harmful versus others	2.4 (1.9–3.0)**	1.6 (1.2–2.1)*	1.4 (1.1–1.9)*	2.6 (2.1–3.3)**	1.3 (0.9–1.7)	1.5 (1.1–2.0)*
**Acceptability of e-cigarettes (or HTPs) use in indoor places**						
Acceptable versus others	3.6 (2.7–4.9)**	2.4 (1.8–3.3)**	2.5 (1.8–3.5)**	3.5 (2.5–4.8)**	2.5 (1.8–3.5)**	2.4 (1.7–3.4)**
**Support for government regulation of e-cigarettes (or HTPs) in the same way as CCs**						
Unsupportive versus others	1.5 (1.1–1.9)*	1.6 (1.2–2.2)**	1.6 (1.2–2.2)*	1.3 (1.0–1.7)*	1.5 (1.1–2.0)*	1.3 (0.9–1.8)

A total of 2971 Koreans were sampled from March 2019 to July 2019. Results are expressed as AOR (95% CI) and p-values are calculated using multivariable analyses after adjusting for sex, age, residential area, educational level, household income, marital status, alcohol consumption frequency, chronic cough, subjective health, survey mode, and the support for government regulation. When assessing the support for government regulation, the harm perception of each product was adjusted instead of the support for government regulation. CC: combustible cigarette. e-cigarette: electronic cigarette. HTP: heated tobacco product. AOR: adjusted odds ratio. CI: confidence interval.

* p<0.05

** p<0.001.

## DISCUSSION

Our results show that current e-cigarette or HTP users had more positive attitudes toward their use than non-e-cigarette or non-HTP users. Compared to non-e-cigarette or non-HTP users, current e-cigarette or HTP users were 4 to 10 times more likely to report that e-cigarette or HTP use is less harmful than CC use with direct or indirect effect. Respondents with more favorable attitudes towards e-cigarettes or HTPs were more likely to accommodate indoor use and less likely to support strict regulation of e-cigarettes or HTPs as CCs. The general harm perceptions and attitudes toward e-cigarettes and HTPs among these tobacco product users appeared similar. Actual use in private or public places was higher in current e-cigarette or HTP users than non-tobacco users or non-e-cigarette (or non-HTP) users and was closely related to their harm perception, attitude toward indoor use, and their position towards having a stricter governmental regulation for e-cigarettes and HTPs as those for CCs.

Tobacco companies promote HTPs as less harmful to health than CCs, although evidence regarding the long-term health effects is still lacking. HTP expose users and bystanders to toxicants at substantially lower levels than CCs^15^. However, Choi et al.^16^ reported that decreased exposure to harmful and potentially harmful constituents does not seem to proportionately reduce HTP-associated health risks.Switching from CCs to HTPs may achieve some risk reduction concerning cardiovascular disease, but that risk remains nevertheless at a high level^16^. On the other hand, several studies have analyzed the potential harm arising from HTPs^17^. The nicotine levels delivered to the aerosol by HTPs were 70-80% those of CCs and higher than those of e-cigarettes. They also contain considerably higher levels of toxins than e-cigarettes. One analysis of HTPs revealed that there were no significant differences in most biomarkers of potential harm between HTP users and cigarette smokers^18^. Other recent studies have suggested possible hepatotoxicity of HTPs and a positive association between ever using HTPs and asthma, allergic rhinitis, and atopic dermatitis among adolescents^19,20^. HTP use was significantly associated with self-reported periodontal diseases and the incidence of an asthma attack in a Japanese study^21,22^. Harm reduction should not equate reduced exposure with reduced risk of severe health effects. Health authorities should demand data from rigorously conducted, randomized, and controlled trials that test the impact of HTPs on meaningful clinical outcomes. Until large-scale pivotal trials provide evidence to the contrary, HTP use is expected to contribute to smoking-related illnesses and should be considered the equivalent of low-grade smoking^17^. However, the marketing by tobacco companies has led to a positive attitude towards HTPs among tobacco product users. Although many smokers understand that e-cigarettes or HTPs are not risk-free, they use e-cigarettes or HTPs due to their belief that there are health benefits over CCs^11^. The positive attitude toward HTPs is one of the independent predictors of initiation or continuation of HTP use. Recent studies from various countries suggest that HTP poly-users may have higher nicotine dependence and are likely to underestimate their harmful effects to a greater extent than HTP-only users^20,23-26^. A recent study of Korean adults showed that HTP-only users and poly-users of tobacco products were more likely to perceive HTPs as less harmful than CCs than cigarette-only smokers^11^.

The actual use of e-cigarettes or HTPs in private or smoke-free public places is influenced by the favorable belief related to e-cigarettes and HTPs and the rigidity of smoke-free policies. The original aim of smoke-free policies was to protect non-smokers from exposure to secondhand smoking. However, e-cigarettes or HTPs have been marketed to smokers, leading smokers to inadvertently circumvent smoke-free policies. The higher social acceptability of e-cigarette or HTP use has made smokers feel more comfortable using them, even in smoke-free public places^27,28^. According to an international tobacco control survey in Australia and the United Kingdom (UK), e-cigarette use in private or public areas is higher in a less restrictive regulatory environment such as the UK than in Australia^27,28^. Japanese studies showed that approximately 20% of e-cigarette users had frequently used e-cigarettes in restaurants and workplaces where cigarette smoking is not allowed^29^, and a considerable number of people used HTPs in locations where tobacco smoking was not allowed^12^. Using e-cigarettes or HTPs in places where cigarette smoking is prohibited could potentially re-normalize tobacco smoking, sustain the poly-use of tobacco products, maintain nicotine addiction, and complicate enforcement of smoke-free policies^30^.

Korean respondents were less likely to think that e-cigarettes are less harmful than CCs (785 out of 2971; 26.4%), compared to UK respondents (800 out of 1419; 56.4%)^27^. Among Korean respondents not using e-cigarettes, more had the view that e-cigarettes were more harmful than CCs (632 out of 1468; 43.1%) than less harmful (265 out of 1468; 18.1%). On the other hand, two-thirds of respondents (65.7%) perceived e-cigarettes as less harmful than CCs, and about half perceived HTPs as less harmful than CCs, among Japanese ITC study respondents^31^. Sutano et al.^32^ reported that HTP use within indoor places was 67.1% in bars and pubs and 37.5% in workplaces, among Japanese HTP users. Another Japanese study excluding participants within permitted venues, found that experience of HTP use was 20.7% at home, 11.8% in restaurants, and 11.9% in workplaces. Our results show that actual use within indoor places among HTP users was 17.4% (246 out of 1416) in public places, 20.4% (289 out of 1416) in their car, and 41.8% (592 out of 1416) at home. In terms of e-cigarette use in indoor places, the UK and Australian data showed that actual use was 27.7% in smoke-free public areas, 56.2% in their car, and 67.9% at home^27^. Interestingly, e-cigarette use within indoor places was more common in Australia, where the regulatory environment for e-cigarettes is more stringent, and vapers who believe e-cigarette use is less harmful than smoking tended to vape more at home and in their car.

### Strengths and limitations

The strengths of the present study are as follows. First, one of the challenges of the studies on e-cigarettes or HTPs is the difficulty of recruiting enough participants due to the lower prevalence of e-cigarette and/or HTP users compared with those of CCs in South Korea. According to the Korean National Health and Nutrition Examination Survey data, the prevalence of current smokers was 36.7% among males and 7.5% among females. The prevalence of e-cigarette (6.3% among males and 0.9% among females) or HTP (7.9% among males and 0.7% among females) use was much lower than cigarette smoking^33^. In particular, the proportions of e-cigarette-only users and dual users of e-cigarettes and HTPs are much lower than 0.2%. Therefore, as in the present study, it is more reasonable to recruit sufficient participants equally for each type of product group by non-probability targeted sampling methods rather than using representative samples^34^. Second, to increase the study’s credibility, we tried to classify tobacco product users accurately by using a screening questionnaire containing pictures of tobacco products and a detailed description of specific brand names. In the meantime, the use of e-cigarettes or HTPs has not been accurately verified in South Korea. HTPs were commonly confused with e-cigarettes since the Korean government categorizes HTPs as ‘cigarette-type e-cigarettes’, the same classification used for e-cigarettes^35,36^. Recent studies in the UK and the US also reported that mentioning brand names of a specific product type or using pictures of tobacco products may improve the accuracy of the assessment^37,38^. Third, after adjusting for multiple potential confounders using data from a large-scale survey, we investigated the association between the attitudes towards e-cigarettes or HTPs and the actual use in private or public places.

However, due to the nature of this study, several limitations should be considered when interpreting the results. First, the samples were not representative of the general population and hence, nationally representative surveys are needed to complement the data. Second, there is a possibility of recruiting a higher income, literate, younger, and more male population than the general population. Third, causality, reverse causality, and temporal relationships could not be ascertained based on cross-sectional data. Further studies using a prospective randomized controlled trial will be needed to determine the causation of our findings. Fourth, data were collected via self-reported surveys and, thus, might have been subject to recall bias and underestimation. Finally, we may not have fully accounted for potential confounders in the analysis.

## CONCLUSIONS

E-cigarette or HTP users were likely to feel that their tobacco products were less harmful than CCs. They were also more inclined to accept indoor use positively but were unlikely to support government regulation of controlling e-cigarettes or HTPs as stringently as CCs, compared to non-e-cigarette or non-HTP users. This attitude towards their tobacco products was associated with their actual use in private and public places where their use is prohibited. There is an urgent need to discuss and introduce more comprehensive tobacco control strategies, including for new tobacco products.

## Data Availability

Data sharing is not applicable to this article as no new data was created.
