# Supplementary material for: Perceived relative harm of heated tobacco products and electronic cigarettes and its association with use in smoke-free places: A cross-sectional analysis of Korean adults
Source: Tob Induc Dis. 2022 Feb 21;20:20. doi: 10.18332/tid/145699 (PMC8859996; doi:10.18332/tid/145699)
Supplement: Supplementary file 1 [file TID-20-20-s1.pdf]

Supplement Table 1 Attitude towards electronic cigarettes (n=2971)

	Non-tobacco (n=167)		CC-only (n=698)		E-cigarette-only (n=316)		HTP-only (n=377)		CC+E-cigarette (n=374)		E-cigarette + HTP (n=303)		CC + HTP (n=393)		Triple (n=343)	
	N	%	N	%	N	%	N	%	N	%	N	%	N	%	N	%
Harmfulness of e-cigarettes (or HTPs) relative to CCs																
Less harmful	22	13.2	110	15.8	127	40.2	85	22.5	145	38.8	116	38.3	70	17.8	110	32.1
Equally harmful	20	12.0	134	19.2	107	33.9	110	29.2	101	27.0	99	32.7	115	29.3	101	29.4
More harmful	105	62.9	330	47.3	76	24.1	145	38.5	113	30.2	83	27.4	157	39.9	123	35.9
Don’t know	20	12.0	124	17.8	6	1.9	37	9.8	15	4.0	5	1.7	51	13.0	9	2.6
Harmfulness of exposure to secondhand aerosol from e-cigarettes (or HTPs) relative to secondhand smoking from CCs																
Less harmful	21	12.6	170	24.4	151	47.8	116	30.8	162	43.3	107	35.3	89	22.6	120	35.0
Equally harmful	29	17.4	139	19.9	97	30.7	102	27.1	95	25.4	95	31.4	109	27.7	95	27.7
More harmful	96	57.5	272	39.0	64	20.3	126	33.4	108	28.9	97	32.0	149	37.9	118	34.4
Don’t know	21	12.6	117	16.8	4	1.3	33	8.8	9	2.4	4	1.3	46	11.7	10	2.9
Acceptability of e-cigarettes (or HTPs) use in indoor places																
Acceptable	3	1.8	70	10.0	60	19.0	61	16.2	70	18.7	48	15.8	51	13.0	69	20.1
Neutral	12	7.2	78	11.2	69	21.8	84	22.3	79	21.1	80	26.4	84	21.4	87	25.4
Unacceptable	145	86.8	475	68.1	182	57.6	210	55.7	216	57.8	169	55.8	224	57.0	179	52.2
Don’t know	7	4.2	75	10.7	5	1.6	22	5.8	9	2.4	6	2.0	34	8.7	8	2.3
Support for government regulation of e-cigarettes (or HTPs) in the same way as CCs																
Unsupportive	24	14.4	106	15.2	95	30.1	94	24.9	104	27.8	97	32.0	83	21.1	103	30.0
Neutral	18	10.8	111	15.9	126	39.9	99	26.3	117	31.3	87	28.7	110	28.0	108	31.5
Supportive	119	71.3	410	58.7	89	28.2	160	42.4	138	36.9	114	37.6	164	41.7	124	36.2
Don’t know	6	3.6	71	10.2	6	1.9	24	6.4	15	4.0	5	1.7	36	9.2	8	2.3

Results are expressed as number (%). Abbreviations: CC, combustible cigarette; e-cigarette, electronic cigarette; HTP, heated tobacco product.

Supplement Table 2 Attitude towards heated tobacco products (n=2971)

	Non-tobacco (n=167)		CC-only (n=698)		E-cigarette-only (n=316)		HTP-only (n=377)		CC+E-cigarette (n=374)		E-cigarette + HTP (n=303)		CC + HTP (n=393)		Triple (n=343)	
	N	%	N	%	N	%	N	%	N	%	N	%	N	%	N	%
Harmfulness of e-cigarettes (or HTPs) relative to CCs																
Less harmful	16	9.6	104	14.9	71	22.5	169	44.8	73	19.5	92	30.4	135	34.4	110	32.1
Equally harmful	21	12.6	124	17.8	91	28.8	106	28.1	127	34.0	88	29.0	116	29.5	95	27.7
More harmful	108	64.7	355	50.9	133	42.1	96	25.5	149	39.8	118	38.9	122	31.0	130	37.9
Don’t know	22	13.2	115	16.5	21	6.6	6	1.6	25	6.7	5	1.7	20	5.1	8	2.3
Harmfulness of exposure to secondhand aerosol from e-cigarettes (or HTPs) relative to secondhand smoking from CCs																
Less harmful	11	6.6	111	15.9	86	27.2	177	46.9	91	24.3	97	32.0	153	38.9	108	31.5
Equally harmful	23	13.8	144	20.6	97	30.7	100	26.5	119	31.8	86	28.4	114	29.0	100	29.2
More harmful	113	67.7	325	46.6	118	37.3	92	24.4	141	37.7	116	38.3	108	27.5	122	35.6
Don’t know	20	12.0	118	16.9	15	4.7	8	2.1	23	6.1	4	1.3	18	4.6	13	3.8
Acceptability of e-cigarettes (or HTPs) use in indoor places																
Acceptable	3	1.8	43	6.2	31	9.8	67	17.8	33	8.8	39	12.9	56	14.2	65	19.0
Neutral	8	4.8	73	10.5	61	19.3	98	26.0	89	23.8	69	22.8	101	25.7	89	25.9
Unacceptable	146	87.4	499	71.5	214	67.7	208	55.2	232	62.0	188	62.0	227	57.8	181	52.8
Don’t know	10	6.0	83	11.9	10	3.2	4	1.1	20	5.3	7	2.3	9	2.3	8	2.3
Support for government regulation of e-cigarettes (or HTPs) in the same way as CCs																
Unsupportive	22	13.2	96	13.8	61	19.3	119	31.6	71	19.0	78	25.7	106	27.0	97	28.3
Neutral	19	11.4	106	15.2	112	35.4	115	30.5	112	29.9	86	28.4	119	30.3	114	33.2
Supportive	118	70.7	422	60.5	132	41.8	137	36.3	170	45.5	132	43.6	154	39.2	129	37.6
Don’t know	8	4.8	74	10.6	11	3.5	6	1.6	21	5.6	7	2.3	14	3.6	3	0.9

Results are expressed as number (%). Abbreviations: CC, combustible cigarette; e-cigarette, electronic cigarette; HTP, heated tobacco product.

© 2022 Kim C.Y. et al.
